# Screening of *CACNA1A* and *ATP1A2* genes in hemiplegic migraine: clinical, genetic, and functional studies

**DOI:** 10.1002/mgg3.24

**Published:** 2013-07-02

**Authors:** Oriel Carreño, Roser Corominas, Selma Angèlica Serra, Cèlia Sintas, Noèlia Fernández-Castillo, Marta Vila-Pueyo, Claudio Toma, Gemma G Gené, Roser Pons, Miguel Llaneza, María-Jesús Sobrido, Daniel Grinberg, Miguel Ángel Valverde, José Manuel Fernández-Fernández, Alfons Macaya, Bru Cormand

**Affiliations:** 1 Departament de Genètica, Facultat de Biologia, Universitat de BarcelonaBarcelona, Spain; 2 Institut de Biomedicina de la Universitat de Barcelona (IBUB)Barcelona, Spain; 3 Center for Biomedical Network Research on Rare Diseases (CIBERER), Institute of Health Carlos IIISpain; 4 Pediatric Neurology Research Group, Vall d'Hebron Research Institute, Universitat Autònoma de BarcelonaBarcelona, Spain; 5 Laboratory of Molecular Physiology and Channelopathies, Department of Experimental and Health Sciences, Universitat Pompeu FabraBarcelona, Spain; 6 First Department of Pediatrics, Agia Sofia Hospital, University of AthensAthens, Greece; 7 Sección de Neurología, Complejo Hospitalario Arquitecto Marcide-Novoa SantosFerrol, Spain; 8 Fundación Pública Galega de Medicina XenómicaSantiago de Compostela, Spain

**Keywords:** *ATP1A2*, *CACNA1A*, functional studies, hemiplegic migraine, mutation analysis

## Abstract

Hemiplegic migraine (HM) is a rare and severe subtype of autosomal dominant migraine, characterized by a complex aura including some degree of motor weakness. Mutations in four genes (*CACNA1A*, *ATP1A2*, *SCN1A* and *PRRT2*) have been detected in familial and in sporadic cases. This genetically and clinically heterogeneous disorder is often accompanied by permanent ataxia, epileptic seizures, mental retardation, and chronic progressive cerebellar atrophy. Here we report a mutation screening in the *CACNA1A* and *ATP1A2* genes in 18 patients with HM. Furthermore, intragenic copy number variant (CNV) analysis was performed in *CACNA1A* using quantitative approaches. We identified four previously described missense *CACNA1A* mutations (p.Ser218Leu, p.Thr501Met, p.Arg583Gln, and p.Thr666Met) and two missense changes in the *ATP1A2* gene, the previously described p.Ala606Thr and the novel variant p.Glu825Lys. No structural variants were found. This genetic screening allowed the identification of more than 30% of the disease alleles, all present in a heterozygous state. Functional consequences of the *CACNA1A*-p.Thr501Met mutation, previously described only in association with episodic ataxia, and *ATP1A2*-p.Glu825Lys, were investigated by means of electrophysiological studies, cell viability assays or Western blot analysis. Our data suggest that both these variants are disease-causing.

## Introduction

Familial or sporadic hemiplegic migraine (FHM, MIM #141599, or SHM) are rare subtypes of migraine with aura (MA) characterized by paroxysmal episodes of hemiparesis generally preceding or accompanying a headache attack (International Headache Society [IHS] 2004)). FHM is considered a monogenic disorder and follows an autosomal dominant inheritance pattern (Pietrobon 2007). Both FHM and SHM are genetically heterogeneous disorders. Up to now, four causative genes have been described in hemiplegic migraine (HM): *CACNA1A* on chromosome 19p13 (FHM1, MIM #301011) (Ophoff et al. 1996), *ATP1A2* at 1q23 (FHM2, MIM #182340) (De Fusco et al. 2003), *SCN1A* at 2q24 (FHM3, MIM #182389) (Dichgans et al. 2005) and, recently, *PRRT2* at 16p11.2 (MIM #614386) (Riant et al. 2012). Additionally, two other *loci* have been reported in FHM families at 1q31 (Gardner et al. 1997) and 14q32 (Cuenca-Leon et al. 2009), although the specific genetic defects have not yet been uncovered.

Mutational screenings of HM patients have reported more than 30 mutations in the *CACNA1A* gene, over 60 mutations in the *ATP1A2* gene, only five in *SCN1A* (de Vries et al. 2009; Riant et al. 2010a; Freilinger et al. 2011) and eight in *PRRT2* (Cloarec et al. 2012; Dale et al. 2012; Gardiner et al. 2012; Marini et al. 2012; Riant et al. 2012). Additionally, a quantitative study that used multiple ligation-dependent probe amplification (MLPA) identified a deletion of exons 39–47 of *CACNA1A* in a SHM patient (Labrum et al. 2009). *CACNA1A* encodes the pore-forming α1 subunit of the voltage-gated neuronal Ca_v_2.1 (P/Q-type) channel. Ca_v_2.1 channels are located in cortical glutamatergic presynaptic terminals and play an important role in controlling neurotransmitter release. *ATP1A2* encodes the α2 subunit of the Na^+^/K^+^ ATPase, is expressed in astrocytes and is involved in the clearance of extracellular K^+^ and production of a Na^+^ gradient used in the reuptake of glutamate. *SCN1A* encodes the α1 subunit of the neuronal voltage-gated sodium channel Na_v_1.1. This channel is critical in the generation and propagation of action potentials (Wessman et al. 2007). Finally, *PRRT2* codes for a transmembrane protein of unknown function that is capable to bind to synaptosomal-associated protein 25 (SNAP25), which suggests a role in synaptic exocytosis (Lee et al. 2012).

The allelic heterogeneity displayed by the *CACNA1A* gene also correlates with substantial clinical variation, as mutations in this gene are also responsible for two other autosomal dominant diseases: episodic ataxia type 2 (EA2, MIM #108500) and spinocerebellar ataxia type 6 (SCA6, MIM #183086). The range of *CACNA1A*-linked phenotypes has even been broadened by the recent descriptions of patients presenting with alternating hemiplegia of childhood (de Vries et al. 2008), acute striatal necrosis (Carreño et al. 2011), hemiplegia–hemiconvulsion–epilepsy syndrome (Yamazaki et al. 2011), and recurrent ischemic stroke (Knierim et al. 2011). Clinical variation is also seen within the HM phenotype, a condition in which *CACNA1A* may sometimes be implicated as a modifier gene rather than a disease-causing gene (Serra et al. 2010). Typical attacks in HM are often associated with other aura symptoms: the clinical spectrum includes permanent cerebellar signs and, less frequently, various types of epileptic seizures, mental retardation, and coma. Furthermore, in approximately 50% of FHM1/*CACNA1A* families, chronic progressive ataxia occurs independently of the migraine attacks (IHS 2004). *ATP1A2* has also been associated with alternating hemiplegia of childhood (Bassi et al. 2004). Also, the *SCN1A* gene has been associated with phenotypes other than HM, as it has been identified as a cause of generalized epilepsy with febrile seizures plus type 2 (GEFS+2, MIM #604403) (Escayg et al. 2000), severe myoclonic epilepsy in infancy (SMEI, MIM #607208), also called Dravet syndrome (Dravet 2011), childhood epilepsy with generalized tonic-clonic seizures (ICEGTC, MIM #607208), familial febrile convulsions type 3A (FEB3A, MIM #604403) (Mantegazza et al. 2005), and elicited repetitive daily blindness (ERDB) with HM (Vahedi et al. 2009). Finally, mutations in *PRRT2* have been found in a number of paroxysmal disorders, including paroxysmal kinesigenic dyskinesia (PKD, MIM #128200), infantile convulsions with PKD (PKD/IC, MIM #602066), benign familial infantile epilepsy (BFIE, MIM #605751), and episodic ataxia or febrile seizures, apart from HM (Wood 2012).

At the functional level, HM and EA2 mutations typically have opposite effects on the Ca_V_2.1 channels leading to increased or decreased Ca^2+^ influx, respectively (Pietrobon 2013). HM-related mutations in the *ATP1A2* gene typically produce a loss of function of the pump (de Vries et al. 2009).

In a previous study, we analyzed 21 Spanish patients with HM episodes and identified three mutations in the *CACNA1A* gene, but no disease-causing changes in *ATP1A2* (Cuenca-Leon et al. 2008). In this study we analyzed 18 additional patients with HM of Spanish and Greek origin and identified four mutations in the *CACNA1A* gene and two mutations in *ATP1A2*. The two changes that had not previously been studied at the functional level were subjected to functional analyses to establish their relevance to the disease phenotype.

## Materials and Methods

### Patients

**Figure 1:**
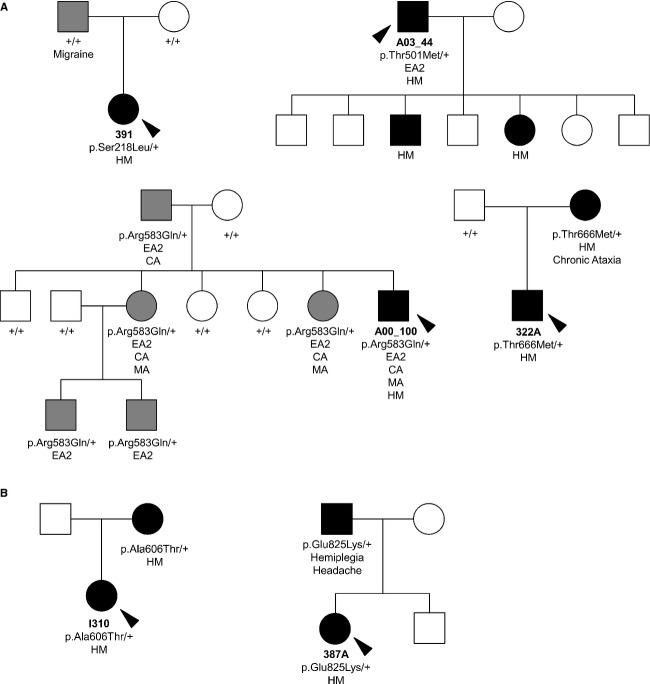
Pedigrees of patients with the identified gene variants. (A) Mutations in the *CACNA1A* gene. (B) Mutations in the *ATP1A2* gene. Affected individuals are denoted by solid symbols; hemiplegic migraine (HM) in black and other phenotypes in gray; squares indicate males and circles females. Probands are indicated by a black arrow. Clinical characteristics are indicated below each individual (HM, migraine with hemiplegic aura; MA, migraine with aura; CA, cerebellar atrophy; EA2, episodic ataxia type 2). Gene variant carrier status is indicated below each patient when known. Mutation p.Ser218Leu appeared de novo in the affected sib.

All 18 patients, examined and diagnosed by specialized neurologists, fulfilled the International Criteria for Headache Disorders 2nd edition (IHS 2004) for FHM or SHM diagnoses except patient #157, with probable HM but only one severe HM episode at the time of diagnosis. Clinical characteristics of the sample are presented in Table 1 and the pedigrees in which mutations were identified are shown in Figure 1. One hundred Caucasian Spanish or Greek unrelated adult control individuals with no personal or family history of recurrent or disabling headache were screened for the presence of the changes identified in the *CACNA1A* and *ATP1A2* genes. The Spanish controls were blood donors or individuals that underwent surgery unrelated to migraine at Hospital Vall d'Hebron (Barcelona), whereas the Greek ones were healthy individuals collected as controls for a mutation screening of cystic fibrosis. This study was approved by the local Ethics Committee and informed consent was obtained from all adult subjects, children, and their parents according to the Helsinki declaration.

**Table 1:** Clinical features of 18 patients with HM and other accompanying symptoms

Patient	Gender	Age (years)	Age at onset (years)	Triggering factors	Diagnosis	Other ictal features	Other	Family history	Origin	Mutation
5C	F	50	11–12	–	FHM	–	–	MA, HM	Spain	–
A03_44	F	66	–	–	FHM	–	Nystagmus, progressive ataxia	HM	Spain	p.Thr501Met (*CACNA1A*)
A00_100	F	31	Adolescent	–	FHM	Episodic ataxia	Progressive ataxia, cerebellar atrophy on MRI	HM, MA, EA2	Spain	p.Arg583Gln (*CACNA1A*)
99	F	40	8	Fasting	SHM	MA	–	MO	Spain	–
112	M	19	11–12	–	SHM	MO	–	Headache	Spain	–
157	M	15	8	Stress	HM (1 episode)	MA	–	MA, MO, vestibular migraine	Spain	–
161	M	20	11	–	SHM		–	–	Spain	–
322B	F	47	<10	–	FHM	MA	Interictal cerebellar signs	HM, interictal nystagmus	Spain	p.Thr666Met (*CACNA1A*)
388A	F	15	11	–	FHM	–	–	HM	Spain	–
391A	F	11	5	Stress	SHM	Generalized seizure, transient cerebral edema on MRI	–	MO	Greece	p.Ser218Leu (*CACNA1A*, de novo)
431	F	42	38	Stress	SHM	–	–	MO	Spain	–
475	F	50	14	–	FHM	–	–	HM	Spain	–
I713	M	37	12	–	FHM	MA	–	HM, MA	Spain	–
G248	F	43	17	Stress, menses	SHM	Aphasia	–	–	Spain	–
8873	F	37	9	–	FHM	Aphasia, transient episodes of bilateral visual loss	–	HM	Spain	–
I310	F	47	15	Stress, strong odors	FHM	Partial epileptic seizures	–	HM	Spain	p.Ala606Thr (*ATP1A2*)
387A	M	10	2	Head injury	FHM	Febrile seizures	Tension-type headache	HM	Greece	p.Glu825Lys (*ATP1A2*)
489A	F	8	5	–	FHM	Prolonged dysphasia/confusion	Nystagmus, cerebellar atrophy on MRI	MA, panic attacks	Spain	–

HM, hemiplegic migraine; FHM, familiar hemiplegic migraine; SHM, sporadic hemiplegic migraine; MA, migraine with aura; MO, migraine without aura; EA2, episodic ataxia type 2; MRI, magnetic resonance imaging.

### Sampling and mutation screening

Peripheral blood samples were collected from all probands. Genomic DNA was isolated using a standard salting-out method (Miller et al. 1988). The *CACNA1A* and *ATP1A2* genes were sequenced as previously described, including all exons, splice sites, and branch points (Cuenca-Leon et al. 2008).

The promoter and 3′UTR regions of *CACNA1A* were also included in the mutational screening. We covered an 894-bp region upstream from the initiation codon and a 553-bp segment including exon 48, as previously described (Veneziano et al. 2009). The extension of the mutational screening was also applied to 18 HM patients from our previous study (Cuenca-Leon et al. 2008). All mutations were assessed by bidirectional sequencing. In addition, all mutations were confirmed by restriction fragment length polymorphism (RFLP) analysis of polymerase chain reaction (PCR) products (Table 2). Control individuals were screened by Sanger sequencing, single-strand conformation polymorphism or RFLP analysis to test the possible presence of the identified changes. Mutation nomenclature follows HGVS guidelines (http://www.hgvs.org/mutnomen/recs-DNA.html) and refers to the *CACNA1A* cDNA sequence NM_023035.1 (protein sequence NP_075461.2), with nucleotide 283A (ATG) corresponding to +1 and the *ATP1A2* cDNA sequence NM_000702.3 (protein sequence NP_000693.1) with nucleotide 1A (ATG) corresponding to the initiation codon.

**Table 2:** Disease-causing mutations identified in the *CACNA1A* and *ATP1A2* genes in hemiplegic migraine patients

		Mutation position							
						
Patient	Gene	Protein	cDNA	Exon	Protein domain	Detection method	Phenotype	Previously reported in other patients	Reference
391A	*CACNA1A*	p.Ser218Leu	c.653C>T	5	Cytoplasmic, IS4-5	−*Taq*I	SHM and generalized seizures	HM	Kors et al. (2001, #164)
A03_44	*CACNA1A*	p.Thr501Met	c.1502C>T	11	Transmembrane, S1 DII	+*Fat*I	FHM, progressive ataxia	EA2	Mantuano et al. (2010, #165)
A00_100	*CACNA1A*	p.Arg583Gln	c.1748G>A	13	Transmembrane, S4 DII	−*Ban*II	FHM, EA2, progressive ataxia	HM	Ducros et al. (2001, #166)
322B	*CACNA1A*	p.Thr666Met	c.1997C>T	17	Transmembrane, hairpin loop DII	+*Bcc*I	FHM, MA	HM	Ophoff et al. (1996, #119)
387A	*ATP1A2*	p.Glu825Lys	c.2473G>A	18	Cytoplasmic loop, L6/7	−*Pvu*II	FHM	No	–
I310	*ATP1A2*	p.Ala606Thr	c.1816G>A	13	Cytoplasmic loop, M4/5	+*Hha*I	FHM, partial epileptic seizures	HM	Riant et al. (2005, #922)

HM, hemiplegic migraine; FHM, familiar hemiplegic migraine; SHM, sporadic hemiplegic migraine; MA, migraine with aura; EA2, episodic ataxia type 2.

Copy number variant (CNV) analysis of *CACNA1A* was performed using two complementary approaches in order to cover most exons (Fig. S1): MLPA and quantitative multiplex PCR of short fluorescent fragments (QMPSF). For the MLPA assay, we used the SALSA MLPA kit P279-A2 for *CACNA1A* (MRC-Holland, Amsterdam, the Netherlands), and for QMPSF we used four sets of probes that covered 16 additional exons not included in the MLPA kit design. Further information about the methods and analysis is provided in Figure S1 and PCR conditions and sample analysis are available upon request.

Paternity was assessed in the de novo mutation identified as previously described (Carreño et al. 2011).

### DNA constructs and site-directed mutagenesis

Human α_1A_ (Ca_V_2.1) was originally cloned into a pCMV vector and mutation p.Thr501Met was introduced by site-directed mutagenesis (GenScript Corporation, Piscatway, NJ). Rabbit α_2_δ and rat Ca_V_β_3_ Ca_V_2.1 channel regulatory subunits were subcloned into a pcDNA3 expression vector.

A full-length human *ATP1A2* ouabain-resistant cDNA clone (α2_oua_wt, with p.Gln116Arg and p.Asn127Asp changes conferring ouabain resistance), housed in pcDNA3.1 with the *myc* tag, was used. Mutation c.2473G>A (α2_oua_825Lys) was introduced in a α2_oua_wt clone with the QuickChange II XL Site-Directed Mutagenesis Kit (Stratagene, La Jolla, CA).

All cDNA clones used in this study were sequenced in full to confirm their integrity.

### Functional analysis of *CACNA1A*

#### Heterologous expression and electrophysiology

HEK 293 cells were transfected using a linear polyethyleneimine (PEI) derivative, the polycation ExGen500 (Fermentas Inc., Hanover, MD) (8 equivalents PEI/3.3 μg DNA/dish) as previously reported (Fernandez-Fernandez et al. 2004). Transfection was performed using the ratio for α_1A_ (wild type or p.Thr501Met), Ca_V_β_3_, α_2_δ, and EGFP (transfection marker) of 1:1:1:0.3. Recordings were done 24–72 h after transfection.

Calcium currents (*I*_Ca_) through wild-type (WT) or p.Thr501Met Ca_V_2.1 (P/Q) channels were measured using the whole-cell configuration of the patch-clamp technique (Hamill et al. 1981). Pipettes had a resistance of 2–3 MΩ when filled with a solution containing (in mmol/L): 140 CsCl, 1 EGTA, 4 Na_2_ATP, 0.1 Na_3_GTP, and 10 Hepes (pH 7.2–7.3 and 295–300 mosmoles/L). The external solution contained (in mmol/L): 140 tetraethylammonium-Cl, 3 CsCl, 2.5 CaCl_2_, 1.2 MgCl_2_, 10 Hepes, and 10 glucose (pH 7.3–7.4 and 300–305 mosmoles/L). Recordings were obtained with a D-6100 Darmstadt amplifier (List Medical, Germany), filtered at 1 kHz and corrected for leak and capacitive currents using the leak subtraction procedure (P/8). Currents were acquired at 33 kHz. The pClamp8 software (Axon Instruments, Foster City, CA) was used for pulse generation, data acquisition, and subsequent analysis.

Peak inward Ca^2+^ currents were measured from cells clamped at −80 mV and pulsed for 20 msec from −60 mV to +70 mV in 5 mV steps. A modified Boltzmann equation (eq. 1) was fitted to normalized current voltage (I–V) to obtain the voltage dependence of activation. The voltage dependence of steady-state inactivation was estimated by measuring peak *I*_Ca_ currents at +20 mV following 30-sec steps to various holding potentials (conditioning pulses) between −80 and +5 mV. During the time interval between test pulses (20 msec) cells were held at −80 mV. *I*_Ca_ currents obtained following the conditioning pulses were normalized to maximal *I*_Ca_ to determine the persistent current. Half-maximal voltage (*V*_1/2, inact_) was obtained by fitting the data to the following Boltzmann equation (eq. 2). All experiments were carried out at room temperature (22–24°C).


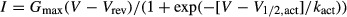
(1)


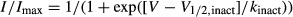
(2)

#### Statistics

Data are presented as the means ± SEM. Student's *t* test or Mann–Whitney test was used for statistical analysis, as appropriate. The *t* test assumes that data are sampled from population that follow a normal distribution and with equal standard deviations. Therefore, the use of the Mann–Whitney test is justified either if at least one of the two population data that are compared fails the normality test (using the Kolmogorov–Smirnov method) or if there are significant differences among the standard deviations of the two populations. Differences were considered significant if *P* < 0.05.

### Functional analysis of *ATP1A2*

#### Cell viability assay

HeLa cells were transfected with 2 μg of constructs using Lipofectamine 2000 (Invitrogen, Carlsbad, CA). Forty-eight hours after transfection, 2/3 of the cells were harvested for Western blot analysis and 1/3 of cells were seeded in petri dishes. After 24 h, the Na^+^/K^+^-ATPase inhibitor ouabain (1 μmol/L) was added to the medium (Dulbecco's modified eagle medium containing 10% fetal calf serum and 1% Penicillin-Streptomycin). After 48 h of ouabain exposition, cell viability was quantified with the 3-(4,5-dimethylthiazol-2-yl)-2,5-diphenyltetrazolium bromide (MTT) reduction assay (De Fusco et al. 2003; Vanmolkot et al. 2003).

#### Western blot analysis

A volume of 25 μg of total protein extract (per lane) was subjected to 10% sodium dodecyl sulfate-polyacrylamide gel electrophoresis. Gels were transblotted to a nitrocellulose membrane and were incubated overnight with monoclonal antibodies against c-Myc (4 μg/mL). Horseradish peroxidase-conjugated anti-mouse immunoglobulins were used as a secondary antibody. Proteins were visualized with the enhanced chemiluminescence kit (GE Healthcare, Little Chalfont, Buckinghamshire, U.K.). Blots were probed with antitubulin antibodies as a loading control.

#### Statistics

We performed four independent experiments, each with triplicate measurements, and carried out statistical analyses by the Student's *t* test, considering one-tail distribution and two-sample equal variance (homoscedastic). Statistical significance was set at *P* < 0.05.

## Results

### Clinical data

Eighteen patients with HM and other accompanying clinical features were identified and screened for mutations in the *CACNA1A* and *ATP1A2* genes. Eleven were classified as FHM and seven as SHM. The clinical characteristics of all patients are summarized in Table 1.

As previously described, we observed a preponderance of female patients in our HM population (13 female patients/five male patients). Age of onset was known for 17 cases. Seven patients presented onset within the first decade; the youngest age of presentation of hemiplegia was at age 2 in one FHM case (#387A). Onset in postpuberty or adolescence was documented in nine patients while in one case (#431) an unusual late onset in the fourth decade was reported. The episodes triggering factors did not differ from those commonly associated with common migraine, except for mild head trauma-induced attacks in case #387A, bearing an *ATP1A2* mutation.

Other ictal manifestations were common in this cohort of HM patients: four cases developed migraine with typical visual or sensory aura and one migraine without aura (MO) episodes; three had episodes of aphasia or dysphasia, presumably the expression of migrainous aura, in the absence of concurrent hemiplegia. Two patients developed epileptic seizures, one partial and one generalized, and one patient had a history of febrile seizures; transient visual loss and episodic ataxia were described in one case each.

Four patients had interictal, permanent neurological abnormalities, mainly a cerebellar syndrome featuring nystagmus and different degrees of chronic ataxia. Three or these harbored mutations in one of the two analyzed genes. All patients were subjected to MRI studies. The only remarkable neuroradiologic findings were chronic cerebellar atrophy in three cases and cerebral edema at the time of the episodes in one case. The remaining patients did not have any chronic disability and had normal interictal exams (Table 1).

A more detailed clinical description of patient #387A and his father, bearing a previously undescribed mutation in the *ATP1A2* gene, is provided as supplementary material (see Data S1).

### Genetic analysis

**Figure 2:**
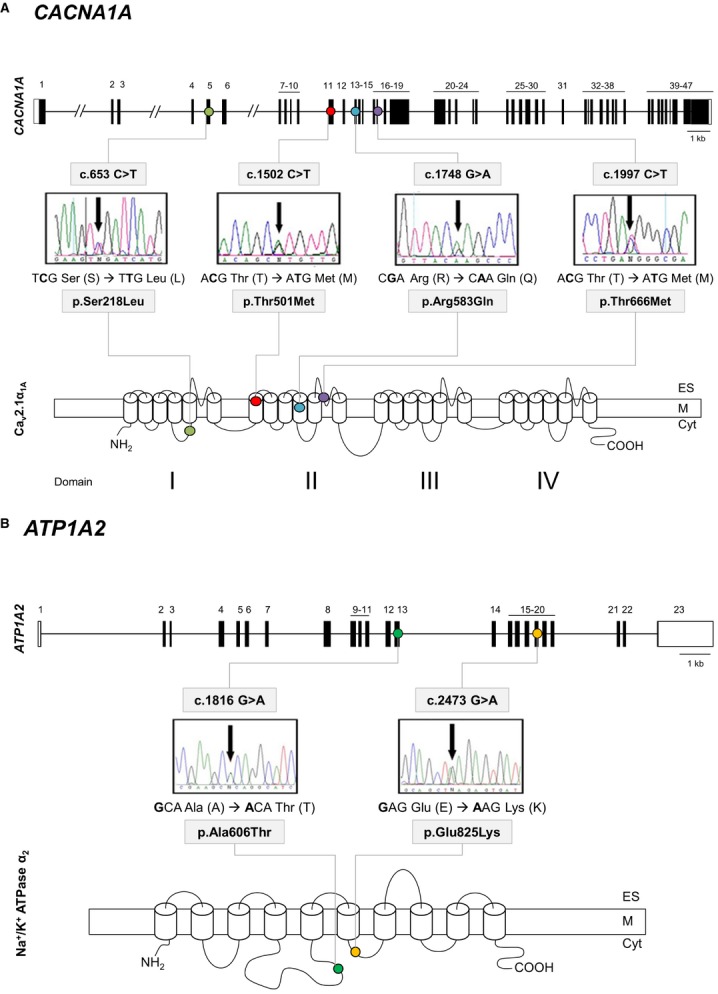
Gene structure of *CACNA1A* and *ATP1A2* and detection of mutations. (A) 1: *CACNA1A* gene structure, with black boxes indicating exons. The identified mutations causing HM are indicated by colored dots: p.Ser218Leu (light green), p.Thr501Met (red), p.Arg583Gln (blue), and p.Thr666Met (purple). 2: Protein structure and location of the identified mutations. (B) 1: *ATP1A2* gene structure, with black boxes indicating exons. The newly identified mutation p.Glu825Lys causing HM is indicated by a yellow dot and p.Ala606Thr with a green dot. 2: Protein structure and location of the identified mutations. Detection of the mutations by direct sequencing of PCR products: electropherograms. Cyt, cytoplasm; M, cytoplasmic membrane; ES, extracellular space.

The extensive sequencing of the *CACNA1A* gene in 18 subjects with HM allowed the identification of four previously reported changes in four unrelated families. In the remaining 14 patients, a novel and a previously described mutation was detected in the *ATP1A2* gene (Table 2 and Fig. 2). The presence of all these variants was confirmed by restriction analysis of the corresponding PCR products (Table 2). The identified changes were not present in 100 Spanish nonmigraineurs. The novel p.Glu825Lys mutation in the *ATP1A2* gene, identified in a Greek patient, was also absent from a set of 100 healthy Greek individuals. One of the changes, p.Ser218Leu, was present in the affected sib, but not in her parents, indicating a de novo origin (Fig. 1A). False paternity was excluded by genotyping 16 polymorphic microsatellite markers in all the family members (data not shown).

All the identified mutations led to amino acid substitutions (p.Ser218Leu, p.Thr501Met, p.Arg583Gln, and p.Thr666Met in *CACNA1A*; p.Glu825Lys and p.Ala606Thr in *ATP1A2*). One of them, p.Glu825Lys in *ATP1A2*, is described here for the first time. The rest of the changes had previously been reported in other patients from several countries, four of them in HM and only one, p.Thr501Met in *CACNA1A*, in association with another phenotype, EA2.

**Figure 3:**
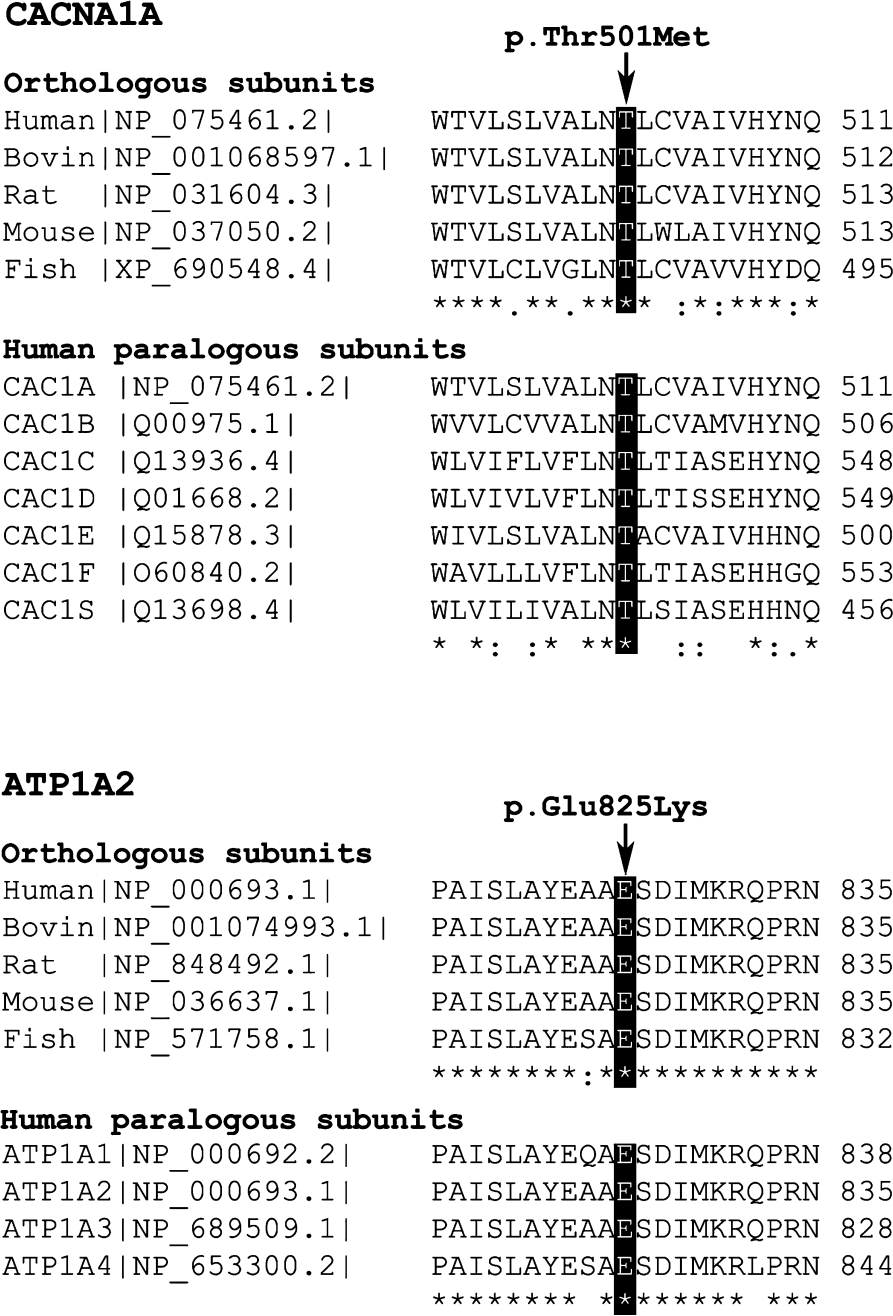
Protein alignment performed with ClustalW (http://www.ebi.ac.uk/Tools/msa/clustalw2) (Chenna et al. 2003). On top, the *CACNA1A* Thr501 residue is conserved in all the human calcium channel α1 subunits studied (CACNA1A, B, C, D, E, F, and S) and in the orthologous CACNA1A proteins of several organisms. Bottom, the *ATP1A2* Glu825 residue is conserved in the four human ATP1 paralogous subunits (ATP1A1, A2, A3, and A4) and in orthologous ATP1A2 proteins of several organisms. Key: “*” identical residues; “:” conserved substitutions (same amino acid group); “.” semi-conserved substitution (similar shapes). Human: *Homo sapiens*; Bovin: *Bos taurus*; Rat: *Rattus norvegicus*; Mouse: *Mus musculus*; Fish (Zebrafish): *Danio rerio*.

The *CACNA1A*-p.Thr501Met mutation, described only once prior to this study, and the novel missense mutation p.Glu825Lys in *ATP1A2*, both of them subjected to functional studies in this study (see below), alter amino acid residues that are highly conserved in evolution as shown by a comparison of their paralogous and orthologous counterparts (Fig. 3). The p.Glu825Lys variant segregates with the HM phenotype, being transmitted from the affected father to the affected sib (Fig. 1B; for a detailed clinical description see Data S1). DNA was not available from the p.Thr501Met family, where a son and a daughter of the studied proband (#A03_44) have also been diagnosed with HM.

The 12 patients with no mutations identified after extensive *CACNA1A* and *ATP1A2* sequencing, as well as 18 HM patients negative for a mutational screening performed in our previous study (Cuenca-Leon et al. 2008), were subjected to MLPA/QMPSF quantitative analysis to seek duplications/deletions in the *CACNA1A* gene. However, no structural variations were found in these probands.

### Functional studies

We subjected two of the identified mutations, p.Thr501Met (*CACNA1A*) and *p.Glu825Lys* (*ATP1A2*), to functional analyses. These changes are the only ones that had not previously been studied by other authors at the functional level.

#### *CACNA1A* (p.Thr501Met): current density and activation/inactivation voltage dependence of heterologously expressed Ca_V_2.1 (P/Q) channels

Mutation p.Thr501Met changes a hydrophilic amino acid to a hydrophobic one and is located in a functionally important region of the human α_1A_ subunit of the neuronal Ca_V_2.1 (P/Q-type) Ca^2+^ channel (Fig. 2A). It lies in the S1 segment of domain II (II-S1) (Fig. 2A), which makes up part of the voltage sensor (Tombola et al. 2006). Because in a previous study of a patient with HM we found that another S1 mutation, p.Tyr1245Cys in domain III of the protein mainly affected voltage dependence of both activation and inactivation of the channel (Serra et al. 2009), we focused our functional analysis of the p.Thr501Met mutation on those same parameters.

**Figure 4:**
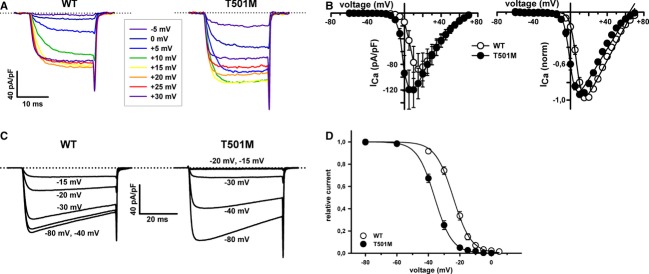
Mutation p.Thr501Met affects activation and inactivation properties of heterologously expressed P/Q channels. (A) Current traces illustrating voltage dependence of WT (left) and p.Thr501Met (right) P/Q channels, in response to 20 msec voltage pulses. Dotted lines in the current traces indicate the zero current level. (B) Current density–voltage relationships (left panels) and normalized I–V curves (right panels) for WT (○) and p.Thr501Met (•) P/Q channels expressed in HEK 293 cells. *V*_1/2, act_ and *k*_act_ values were (in mV): WT (○, *n* = 9) 7.1 ± 0.8 and 3.3 ± 0.3; p.Thr501Met (•, *n* = 14) −0.04 ± 0.99 and 2.8 ± 0.3, respectively. No significant difference was found for *k*_act_ values (*P* = 0.29). (C and D) Steady-state inactivation of WT or p.Thr501Met P/Q channels. Amplitudes of currents elicited by test pulses to +20 mV were normalized to the current obtained after a 30-sec prepulse to −80 mV and fitted by a single Boltzmann function (see Materials and Methods, eq. 2). *V*_1/2, inact_ and *k*_inact_ values were (in mV): WT (○, *n* = 10) −24.2 ± 0.9 and −5.5 ± 0.4; p.Thr501Met (•, *n* = 15) −35.9 ± 1 and −5 ± 0.2, respectively. No significant difference was found for *k*_inact_ values (*P* = 0.53).

Maximum current densities resulting from expression of mutant p.Thr501Met α_1A_ (α_1A(T501M)_) were significantly higher than current densities of wild-type (WT) α_1A_ channels (*P* < 0.05) in a physiological range of depolarized voltages (from −15 to +5 mV) (Fig. 4A and B, left panel). The potential for half-maximal activation (*V*_1/2, act_) was also significantly (*P* < 0.0001) shifted to hyperpolarized potentials for p.Thr501Met channels (by ∼7 mV) (Fig. 4B, right panel). Consistently, the maximum current amplitude was elicited by depolarizing pulses to ∼ +15 mV or ∼ +5 to +10 mV for WT or p.Thr501Met channels, respectively (Fig. 4A and B). The half-maximal voltage for steady-state inactivation (*V*_1/2, inact_) induced by 30-sec-conditioning prepulses between −80 and +5 mV was left-shifted (∼12 mV) in p.Thr501Met channels (*P* < 0.0001). (Fig. 4C and D).

#### *ATP1A2* (p.Glu825Lys): Ouabain resistance survival assays

p.Glu825Lys exchanges a negatively charged residue for a positive one and is located in the intracellular L6/7 loop of the ATP1A2 protein, between the transmembrane segments M6 and M7 (Fig. 2B). Potential pathogenicity of the mutation p.Glu825Lys was indirectly tested through ouabain resistance survival assays.

**Figure 5:**
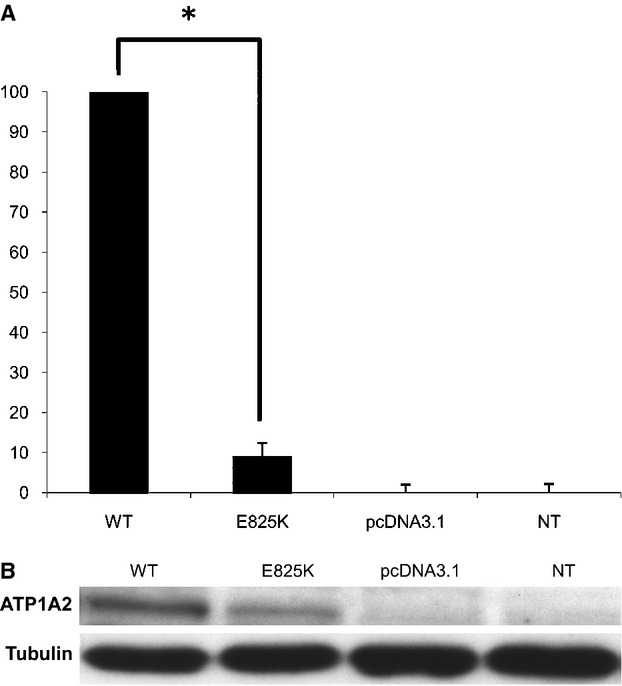
(A) Viability of HeLa cells transfected with the mutant *ATP1A2* cDNA (E825K) normalized to the viability of cells transfected with the ouabain-resistant wild-type *ATP1A2* cDNA (WT). NT, untransfected cells; pcDNA3.1, cells transfected with the empty vector. Four independent experiments were performed, each with triplicate measurements. The * symbol indicates the existence of significant differences between the p.Glu825Lys and the WT *ATP1A2* constructs (*P* < 0.0003). (B) Western blot assay of the different protein extracts using anti-myc and anti-tubulin antibodies. The molecular weight of the Na^+^/K^+^-ATPase α2 subunit and tubulin are indicated. The constructs with the *ATP1A2* ouabain-resistant cDNA carry the myc tag. The clone with the mutation displayed diminished band intensity.

HeLa cells transfected with the α2_oua_825Lys construction showed a 10% survival rate, compared with cells transfected with α2_oua_ WT (*P* < 0.0003) (Fig. 5A). Western blot analysis of HeLa extracts obtained after transfection with the mutant and WT constructions showed a diminished amount of 825Lys protein compared with the WT, indicating that the altered protein may be unstable (Fig. 5B).

## Discussion

We have identified six potentially disease-causing missense mutations in the *CACNA1A* and *ATP1A2* genes in a cohort of 18 unrelated probands with HM from Spain and Greece (33% of patients covered).

There is evidence supporting the finding that the identified amino acid substitutions are indeed disease-causing mutations: (1) When family material was available, the mutations cosegregated with the disease phenotype within the families, (2) the changes were not present in at least 200 chromosomes from unaffected individuals nor in the main public single-nucleotide polymorphism databases (Hapmap, dbSNP), (3) no other molecular alterations were identified within the studied genes after the analysis of the whole coding region and the exon–intron boundaries, including splice sites, branch points, the promoter, and the 3′UTR region in *CACNA1A*, (4) for the two mutations subjected to functional studies the involved amino acid residues are strongly conserved in evolution, both at the intraspecific (CACNA1A, B, C, D, E, F, S or ATP1A1, A2, A3, A4) and interspecific levels (CACNA1A or ATP1A2 subunits from human, cattle, rat, mouse and zebrafish), indicating functional/structural relevance, (5) all these mutations but one (p.Glu825Lys) have been reported previously in other HM or EA2 cohorts, and (6) functional analyses reported here (p.Thr501Met in *CACNA1A* and p.Glu825Lys in *ATP1A2*) and by other authors (p.Ser218Leu [Tottene et al. 2005; Weiss et al. 2008; Debiais et al. 2009; Adams et al. 2010], p.Arg583Gln [Kraus et al. 2000] and p.Thr666Met [Kraus et al. 1998; Tottene et al. 2002] in *CACNA1A*; p.Ala606Thr [Jen et al. 2007; Tavraz et al. 2008] in *ATP1A2*) demonstrate functional consequences of all six changes suggesting pathogenicity.

Interestingly, all but one of the patients that have HM plus additional ictal/interictal neurological features or atypical paroxysmal signs (e.g., epilepsy or cerebral edema on MRI) bore mutations in one of the two genes studied. In general, this was not the case for patients without such “extended” HM phenotype, with no mutations identified in those genes. The molecular defects associated with this extended phenotype are unlikely to mimic those present in common migraine. It is, however, possible that HM cases without known mutation and with less severe clinical presentations, which often overlap with common migraine (in our series most had MO and MA attacks in addition to HM) may represent a better model for primary headaches. In this regard, deciphering the molecular basis of this group of patients may shed new light on the global landscape of migraine genetics.

### *CACNA1A* screening

Four patients presented missense mutations in the *CACNA1A* gene, all of them described in previous screenings by other authors: p.Ser218Leu, p.Arg583Gln, p.Thr666Met, and p.Thr501Met (Fig. 2A).

#### p.Ser218Leu

This change was identified as a *de novo* mutation in a SHM patient with aphasia, transient cerebral edema and generalized seizures. This transition in a CpG dinucleotide had been described in eight HM patients (Kors et al. 2001; Curtain et al. 2006; Chan et al. 2008; Debiais et al. 2009; Stam et al. 2009; Zangaladze et al. 2010) having appeared de novo in two of them (Stam et al. 2009). Previous electrophysiological studies for the p.Ser218Leu mutation revealed a lower activation threshold, an increase of channel recovery after inactivation (Tottene et al. 2005) and the reduction of the inhibitory pathway carried by G-protein-coupled-receptor activation (Weiss et al. 2008). This dramatic gain of function, compared to other missense changes, is thought to explain the severe clinical outcome observed in the patients carrying this mutation. Studies with a mouse model provide evidence that p.Ser218Leu mutations directly affect Ca^2+^-dependent facilitation and synaptic plasticity (Adams et al. 2010).

#### p.Arg583Gln

This prevalent mutation has been previously identified in FHM families and in sporadic patients (Battistini et al. 1999; Ducros et al. 2001; Terwindt et al. 2002;Alonso et al. 2003; Thomsen et al. 2007; Riant et al. 2010a). In family #A00_100, only the proband presents HM (Fig. 1A) and the mutation cosegregates with EA2, cerebellar atrophy and/or HM. Previous functional studies for the p.Arg583Gln mutation revealed a change in the voltage dependence of activation and inactivation toward more negative potentials due to the neutralization of residue 583 that is positively charged in the WT channel, and a decrease in the recovery of inactivation (Kraus et al. 2000).

#### p.Thr666Met

This is also a prevalent mutation that has previously been found in 24 FHM or SHM probands (Ophoff et al. 1996; Ducros et al. 1999; Friend et al. 1999; Terwindt et al. 2002; Wada et al. 2002; Kors et al. 2003; Thomsen et al. 2007; Freilinger et al. 2008; Yabe et al. 2008; Riant et al. 2010a) Functional studies in heterologous systems showed a gain of function through the following mechanisms: a decrease in the recovery after inactivation (Kraus et al. 1998) and increased Ca^2+^ influx through the channel in a broad voltage range around the threshold of activation, which was also reduced (Tottene et al. 2002).

#### p.Thr501Met

This change, found in patient #A03_044 with FHM, nystagmus, and episodic and progressive ataxia, has been described in another family with EA2 (Mantuano et al. 2010) and in a case with bouts of episodic ataxia and confusion but no hemiplegia, in association with vermian cerebellar atrophy (Cleves et al. 2009). This is the second reported mutation in the S1 segment in any domain of the α_1A_ subunit in patients with HM. As the previously described p.Tyr1245Cys (Serra et al. 2009), p.Thr501Met alters channel activation and inactivation. It promotes channel activity by shifting the current activation curve to lower voltages (∼9 mV) and increasing Ca^2+^ current density to a range of voltages that neurons can encounter during action potential firing. p.Thr501Met also shifts voltage-dependent steady-state inactivation to less depolarized voltages (∼15 mV). These results further support an important role of the S1 segments in the function of voltage sensors leading to channel gating (Campos et al. 2007).

The functional consequences of mutation p.Thr501Met are consistent with a causative role in the disease. In this respect, a reduction in the voltage threshold of channel activation by ∼10 mV is a trait shared by all FHM-causing mutations in *CACNA1A* (van den Maagdenberg et al. 2004; Tottene et al. 2005). Such gain of channel function specifically promotes cortical excitatory neurotransmission and favors cortical spreading depression (CSD) initiation and propagation in FHM knock-in (KI) mouse models (Tottene et al. 2009), which has been pointed out as the cause of the aura and migraine itself (Bolay et al. 2002; Pietrobon 2007).

Mutations in *CACNA1A* are also associated with other autosomal dominant neurological disorders characterized by cerebellar dysfunction, such as EA2 (Ophoff et al. 1996). However, contrary to FHM mutations, most EA2 mutations produce loss-of-channel function (Strupp et al. 2007). Our patient carrying the p.Thr501Met CACNA1A mutation also developed cerebellar symptoms. This also occurred with other FHM-causing *CACNA1A* mutations (Ducros et al. 2001). As yet, it is not clear why some *CACNA1A* mutations cause pure FHM and other FHM with cerebellar signs, as functional studies in vitro do not reveal any notable difference among these two groups of FHM mutations (Pietrobon 2007). Nevertheless, the study of p.Arg192Gln and p.Ser218Leu KI animals may help us to unveil the role of FHM *CACNA1A* mutations in EA2 (van den Maagdenberg et al. 2004, 2010). While homozygous p.Arg192Gln (RQ/RQ) and heterozygous p.Ser218Leu (SL/WT) mice did not exhibit an overt phenotype, homozygous p.Ser218Leu (SL/SL) KI model exhibited the main features of the severe p.Ser218Leu clinical syndrome, including mild permanent cerebellar ataxia (van den Maagdenberg et al. 2004, 2010).

### *ATP1A2* screening

#### p.Ala606Thr

This mutation was found in a patient with FHM and focal epileptic seizures. It has previously been reported in three FHM families (Jen et al. 2007; Riant et al. 2005). Functional assays on HeLa cells transfected with WT and mutant *ATP1A2* constructs suggested a loss of function of the sodium–potassium pump (Jen et al. 2007). Same results were found for mutations p.N717K and p.P786L, also located in the L4/5 cytoplasmatic loop of the protein (Jen et al. 2007; Tavraz et al. 2008). A more detailed electrophysiological functional study showed that the Na^+^/K^+^-ATPase activity was decreased due to a lower affinity for potassium (Tavraz et al. 2008).

#### p.Glu825Lys

This novel change, located in the L6/7 loop (Fig. 2B), was identified in an individual with HM and seizures. Ouabain-resistant survival assays showed a decrease in cell viability in HeLa cells transfected with the mutant *ATP1A2* construct (Fig. 5A), again supporting the idea that the mutation results in loss of function of the sodium–potassium pump. Also, Western blot analysis suggests that the mutant protein may be unstable. Other mutations in this loop that have been identified by others and were functionally tested are as follows: p.Met829Arg, p.Arg384*, and p.Arg834Gln (de Vries et al. 2007; Tavraz et al. 2008). p.Arg834* showed no cell survival in ouabain challenge assays, and electrophysiological analyses of p.Arg834Gln displayed altered affinities for extracellular cations or reduced enzyme turnover. It was suggested that negatively charged residues in loop L6/7 contribute to the initial recognition of Na^+^ or K^+^ ions and constitute the cytoplasmic cation entry port (Shainskaya et al. 2000; Jorgensen et al. 2003). Other experiments found that loop L6/7 is important in the transmission of the activation signal initiated by cation binding to the phosphorylation domain of the protein (Xu et al. 2003). These experiments included a Glu to Ala mutation in a Ca^2+^-ATPase that is equivalent to the p.Glu825Lys identified in our patient.

### Unidentified mutations/genes

Overall, 12 of 18 HM patients included in this study do not bear mutations in either *CACNA1A* or *ATP1A2*. Considering also the HM patients screened by us in a previous study (Cuenca-Leon et al. 2008), the level of molecular identification is 9/39 (23%), suggesting the involvement of other genes that still need to be uncovered. In sum, from these 39 patients, 22 were FHM (eight bearing mutations, 36%) and 17 were SHM (only one identified mutation, 6%). These data are in line with previous studies where the coverage was around 40% in FHM patients (Riant et al. 2005) and between 7% and 16% in SHM (Terwindt et al. 2002; de Vries et al. 2007), indicating that *CACNA1A* and *ATP1A2* are major genes in the familial forms of HM but not in the sporadic ones, where there might be a larger genetic heterogeneity and/or other contributing factors. Only in one study with SHM patients presenting an early-onset of the disease the coverage was higher when analyzing the *CACNA1A*, *ATP1A2*, and *SCN1A* genes (Riant et al. 2010a). It is also possible that a few pathogenic mutations have remained unidentified in the two genes studied, including changes in introns or in distant regulatory regions, or CNVs in *ATP1A2* that may be undetectable by PCR. Previously, a large rearrangement in the *CACNA1A* gene was described in a SHM patient (Labrum et al. 2009), and so it is conceivable that deletions or duplications in *ATP1A2* could also be responsible for the disorder. However, CNV analysis has only reported this single deletion in *CACNA1A* so far in HM, and this type of alteration is more frequently found in episodic ataxia, where the typical pathogenic mechanism is a loss of function of the channel (Riant et al. 2008, 2010b; Labrum et al. 2009; Wan et al. 2011).

Mutations in *SLC4A4* have been reported in pedigrees with proximal renal tubular acidosis (pRTA) with HM and migraine (Demirci et al. 2006; Suzuki et al. 2010), but this gene was not considered in this study as none of the patients in our series showed renal abnormalities. Mutations in the *SCN1A* gene, previously linked to FHM and involved in several forms of epilepsy, would appear to be a rather unusual cause of HM. Indeed, more than 700 mutations have been identified in *SCN1A* with SMEI compared to just five in FHM (http://www.molgen.vib-ua.be/SCN1AMutations) (Claes et al. 2009). Furthermore, very recently the genetic heterogeneity of HM phenotype has been broadened with the *PRRT2* gene, previously related to paroxysmal kinesigenic dyskinesia and other episodic disorders. Eight mutations have been reported in 249 screened HM cases (Cloarec et al. 2012; Dale et al. 2012; Gardiner et al. 2012; Marini et al. 2012; Riant et al. 2012). Both *SCN1A* and *PRRT2* may be targeted by sequencing in the future, although they are not expected to explain a substantial proportion of our unresolved HM cases.

Finally, at least one other FHM locus has been mapped out of the three known *loci*, at 14q32, but the underlying gene still awaits identification (Cuenca-Leon et al. 2009). It is very likely that most of the unresolved 12 HM patients from the present cohort and 18 HM patients from a previous study by our group (Cuenca-Leon et al. 2008) bear mutations in other yet unknown HM genes that need to be uncovered. Thus, future studies will use whole-exome sequencing to find new genes responsible for the disease.

## Supporting Information

**Figure S1.** CNV study design using multiplex ligation-dependent probe amplification (MLPA) and quantitative multiplex PCR of short fluorescent fragments (QMPSF) approaches. Symbols (▲) indicate the exons covered by each assay. The MLPA kit allowed inspection of 24 exons. The analysis software Coffalyser v8 was used to evaluate the possible presence of CNVs, considering a deletion when the ratio was under 0.7 and a duplication when it was over 1.3. For QMPSF, we used four sets of primer pairs covering 16 additional exons. A reference fragment from the *RNF20* gene was coamplified in each multiplex. Furthermore, two individuals with deletions corresponding to exons 20–47 and 32–47 of *CACNA1A* were also included in every assay as positive controls. Data were analyzed using the PeakScanner™ v1.0 software (Applied Biosystems), and the final ratios for each exon were expressed using the following formula: (height of the peak corresponding to the tested fragment for the patient/height of the peak corresponding to *RNF20* for the patient)/(height of the peak corresponding to the tested fragment for the average of controls/height of the peak corresponding to *RNF20* for the average of controls). We considered a deletion when the ratio was under 0.6 and a duplication when it was over 1.4.

**Data S1.** Clinical features of patient #387A and his father.
