# Supplementary material for: Screening of CACNA1A and ATP1A2 genes in hemiplegic migraine: clinical, genetic, and functional studies
Source: Mol Genet Genomic Med. 2013 Jul 2;1(4):206–22. doi: 10.1002/mgg3.24 (PMC3865589; doi:10.1002/mgg3.24)
Supplement: Supplementary file 1 [file mgg30001-0206-SD1.doc]

## SUPPLEMENTARY MATERIALS AND METHODS

### Clinical features of patient #387A and his father

This patient is an 8-years old Greek boy who developed simple febrile seizures at ages 11 and 14 months. He has suffered 4 paroxysmal neurological episodes precipitated by mild accidental head trauma without loss of consciousness.

The first episode was at 2 years of age. After the head trauma he was irritable and screaming but could stand or walk. Focal weakness was not reported. He presented right-sided numbness, had difficulty to walk and speak and the symptoms rapidly evolved into right-sided weakness, inability to talk and severe feeling of pain all over the head associated with photophobia, but no nausea or vomiting. All the episodes lasted 0.5 to 1 hour. Since age 7 years he suffers from frequent tension-type headaches, associated with abdominal discomfort.

His 46-year old father suffers since age 7 from 1-2 episodes per year of dizziness followed by dysarthria and right-sided hemiplegia accompanied by severe headache. Sometimes he refers photophobia and nausea after the attack. He was diagnosed with epilepsy in childhood and is currently being treated with valproic acid. His EEG has shown epileptiform discharges.

**Supplementary Figure 1**

CNV study design using Multiplex Ligation-dependent Probe Amplification (MLPA) and Quantitative Multiplex PCR of Short fluorescent Fragments (QMPSF) approaches. Symbols (▲) indicate the exons covered by each assay. The MLPA kit allowed inspection of 24 exons. The analysis software Coffalyser v8 was used to evaluate the possible presence of CNVs, considering a deletion when the ratio was under 0.7 and a duplication when it was over 1.3. For QMPSF, we used four sets of primer pairs covering 16 additional exons. A reference fragment from the *RNF20* gene was co-amplified in each multiplex. Furthermore, two individuals with deletions corresponding to exons 20-47 and 32-47 of *CACNA1A* were also included in every assay as positive controls. Data were analyzed using the PeakScanner^TM^ v1.0 software (Applied Biosystems), and the final ratios for each exon were expressed using the following formula: (height of the peak corresponding to the tested fragment for the patient /

height of the peak corresponding to *RNF20* for the patient) / (height of the peak

corresponding to the tested fragment for the average of controls / height of the peak

corresponding to *RNF20* for the average of controls). We considered a deletion when the ratio was under 0.6 and a duplication when it was over 1.4.
