# Supplementary figures and images for: Screening of CACNA1A and ATP1A2 genes in hemiplegic migraine: clinical, genetic, and functional studies
Source: Mol Genet Genomic Med. 2013 Jul 2;1(4):206–22. doi: 10.1002/mgg3.24 (PMC3865589; doi:10.1002/mgg3.24)

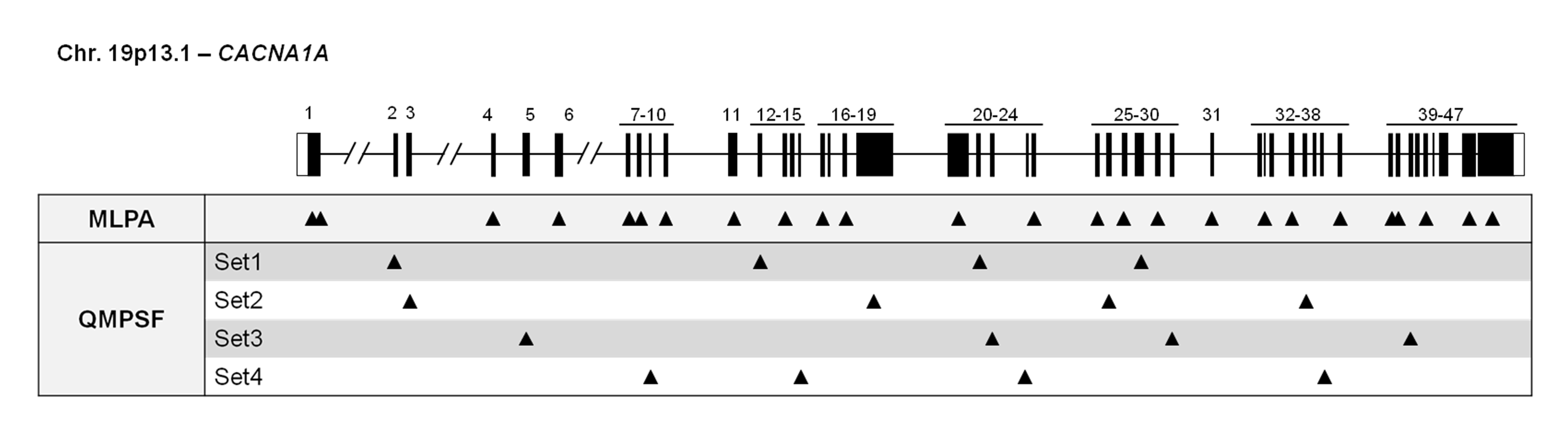

Supplement: Supplementary file 2 [file mgg30001-0206-SD2.tif]
